# Supplementary figures and images for: Independent genomic polymorphisms in the PknH serine threonine kinase locus during evolution of the Mycobacterium tuberculosis Complex affect virulence and host preference
Source: PLoS Pathog. 2020 Dec 21;16(12):e1009061. doi: 10.1371/journal.ppat.1009061 (PMC7785237; doi:10.1371/journal.ppat.1009061)

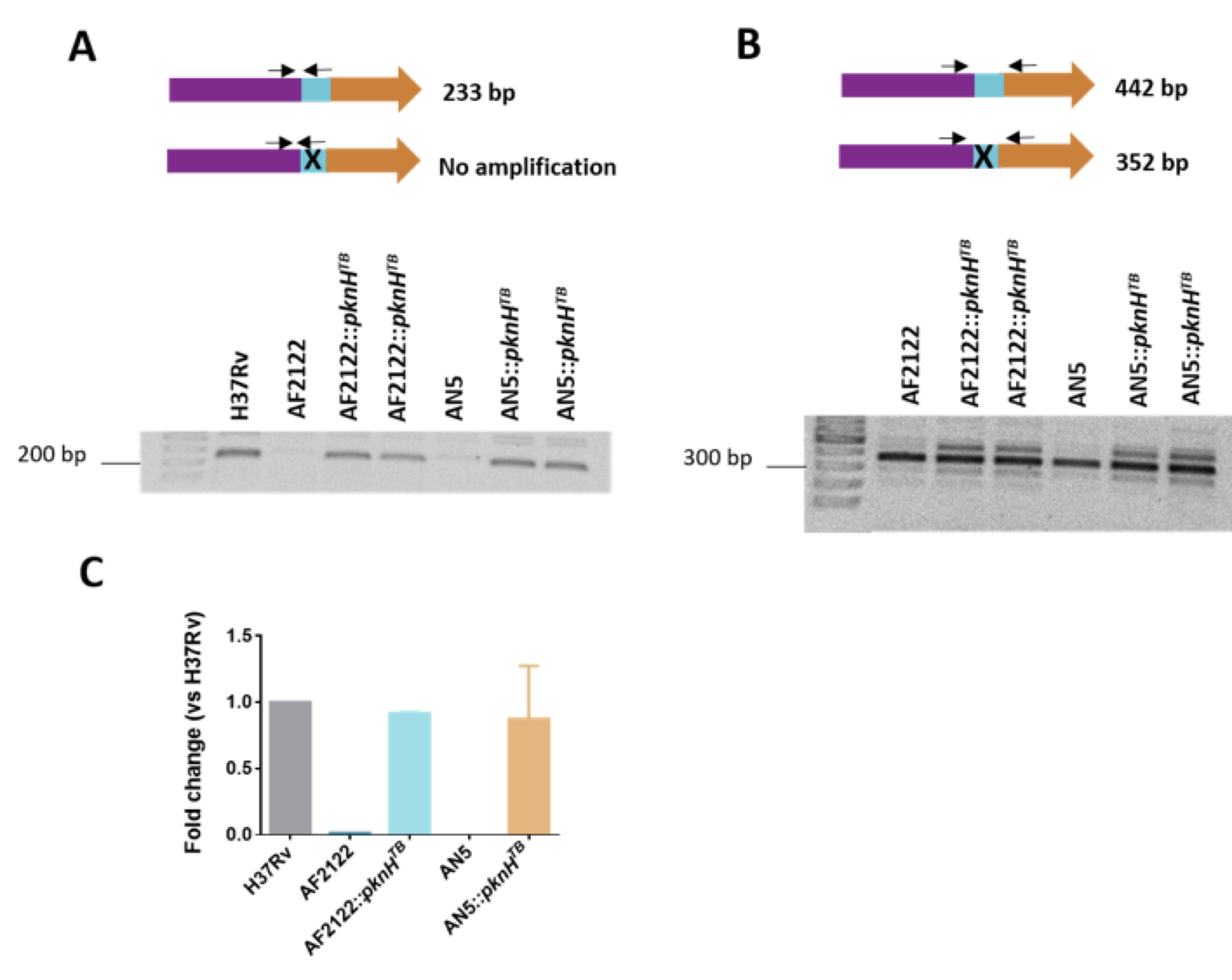

Supplement: S1 Fig — (A, B) Confirmation of pknHTB presence in M. bovis::pknHTB strains. cDNA was analyzed by PCR, either with the reverse primer included inside the proline-rich region (A), or with both primers flanking this sequence (B). In the first case, cDNA amplification was only observed when pknHTB was expressed, whereas in the second case amplification occurred in both cases, with a different fragment size depending on the pknH expressed. (C) pknHTB expression was measured by qRT-PCR using the proline-rich region flanking primers. Graph represents mean±SD from one experiment with three replicates, corresponding to the fold-change value in comparison to H37Rv expression. (TIF) [file ppat.1009061.s001.tif]

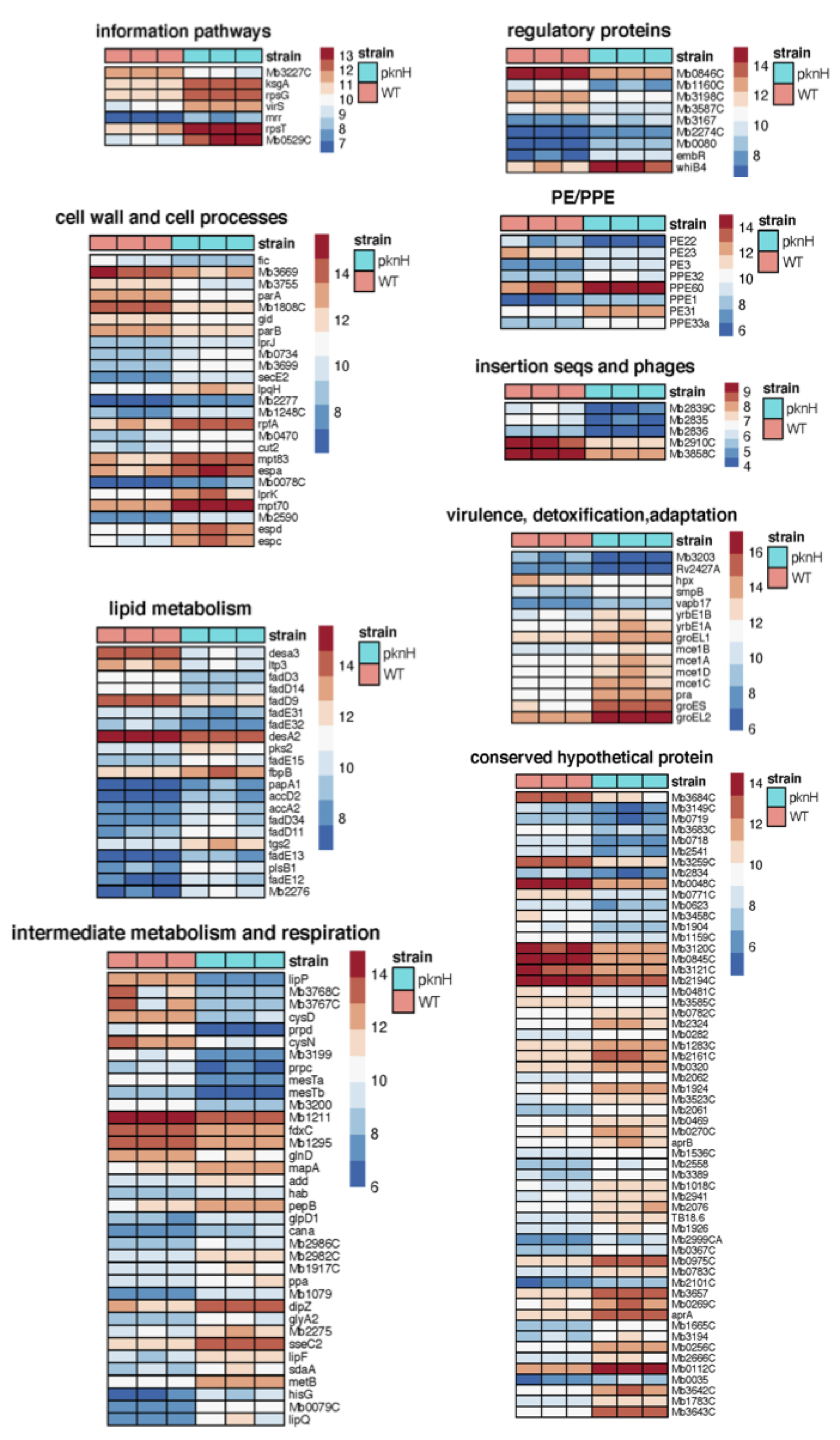

Supplement: S2 Fig — (TIF) [file ppat.1009061.s002.tif]

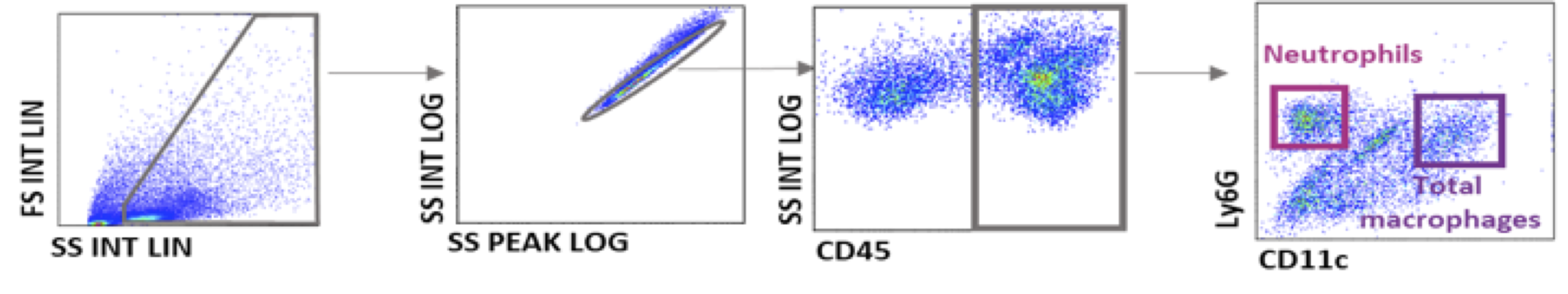

Supplement: S3 Fig — (TIF) [file ppat.1009061.s003.tif]
