# Supplementary material for: Independent genomic polymorphisms in the PknH serine threonine kinase locus during evolution of the Mycobacterium tuberculosis Complex affect virulence and host preference
Source: PLoS Pathog. 2020 Dec 21;16(12):e1009061. doi: 10.1371/journal.ppat.1009061 (PMC7785237; doi:10.1371/journal.ppat.1009061)
Supplement: S1 Table — (DOCX) [file ppat.1009061.s004.docx]

**Table S1. Genomic data of MTBC isolates used for the genomic analysis of RD900 locus.**

| **ACCESSION** | **LINEAGE** | **ORIGIN** | **REFERENCE** |
| --- | --- | --- | --- |
| ERR015598 | *M. canetti* (CIPT140010059) STB-A | NA | Wellcome Sanger Institute |
| SRR011186 | *M. canetii* | NA | Broad Institute |
| ERR1336826 | *M. canetii* | NA | Institut Pasteur |
| ERR313114 | *M. canetii* | Djibouti | Blouin et al., 2014 |
| SRR6650709 | *M. canetti* (NYS IDR1300035880) | NA | New York State Department of Health |
| ERR1109376 | *M. canetti* (CIPT 140070010) STB-K-S | NA | European Molecular Biology Laboratory |
| ERR1109377 | *M. canetti* (CIPT 140070010) STB-K-R | NA | European Molecular Biology Laboratory |
| ERR233356 | *M. tuberculosis* L1 | USA | Comas et al., 2013 |
| ERR1200629 | *M. tuberculosis* L1 | Ethiopia | Comas et al., 2013 |
| ERR234155 | *M. tuberculosis* L1 | Germany | Comas et al., 2013 |
| ERR234238 | *M. tuberculosis* L1 | Vietnam | Comas et al., 2013 |
| ERR234272 | *M. tuberculosis* L1 | UK | Comas et al., 2013 |
| ERR233377 | *M. tuberculosis* L1 | Nepal | Centre for Public Health Research, University of Valencia |
| ERR1200603 | *M. tuberculosis* L7 | Ethiopia | Comas et al., 2013 |
| ERR1200617 | *M. tuberculosis* L7 | Ethiopia | Comas et al., 2013 |
| ERR1200635 | *M. tuberculosis* L7 | Ethiopia | Comas et al., 2013 |
| ERR1200640 | *M. tuberculosis* L7 | Ethiopia | Comas et al., 2013 |
| ERR234098 | *M. tuberculosis* L2 | China | Comas et al., 2013 |
| ERR233386 | *M. tuberculosis* L2 | India | Comas et al., 2013 |
| ERR015616 | *M. tuberculosis* L2 | Russia | Comas et al., 2013 |
| ERR233391 | *M. tuberculosis* L3 | Nepal | Comas et al., 2013 |
| ERR233360 | *M. tuberculosis* L3 | USA | Comas et al., 2013 |
| ERR234153 | *M. tuberculosis* L3 | Germany | Comas et al., 2013 |
| ERR233358 | *M. tuberculosis* L4 | USA | Comas et al., 2013 |
| ERR1200614 | *M. tuberculosis* L4 | Ethiopia | Comas et al., 2013 |
| ERR233373 | *M. tuberculosis* L4 | UK | Comas et al., 2013 |
| ERR233379 | *M. tuberculosis* L4 | Nepal | Comas et al., 2013 |
| ERR234154 | *M. tuberculosis* L4 | Germany | Comas et al., 2013 |
| ERR234097 | *M. tuberculosis* L5 | Germany | Comas et al., 2013 |
| ERR234199 | *M. tuberculosis* L5 | Ghana | Comas et al., 2013 |
| ERR017801 | *M. tuberculosis* L5 | Russia | Casali et al., 2014 |
| ERR233366 | *M. tuberculosis* L6 | Gambia | Comas et al., 2013 |
| ERR234184 | *M. tuberculosis* L6 | Germany | Comas et al., 2013 |
| ERR400537 | *M. tuberculosis* L6 | UK | Walker et al., 2015 |
| SRR998594 | *M. tuberculosis* L6 | Mali | Broad Institute |
| ERR125602 | *M. bovis* | UK | Trewby et al., 2016 |
| SRR5216693 | *M. bovis* | New Zealand | Crispell et al., 2017 |
| ERR551009 | *M. bovis* | Germany | Walker et al., 2015 |
| ERR552138 | *M. bovis* | Republic of the Congo | Malm et al., 2017 |
| ERR400386 | *M. bovis* | UK | Walker et al., 2015 |
| WT_S18_L001-4 | *M. bovis* AF2122/97 | Laboratory strain | This study |
| SRR1173570 | *M. bovis* BZ | Uganda | Wanzala et al., 2015 |
| SRR1173284 | *M. bovis* B2 | Uganda | Wanzala et al., 2015 |
| SRR022532 | *M. bovis* Ravenel | Laboratory strain | Waters et al., 2009 |
| ERR017778 | BCG | Russia | Casali et al., 2014 |
| ERR161048 | BCG | Malawi | Guerra-Assuncao et al., 2015 |
| ERR234151 | BCG | Laboratory BCG strain | Comas et al., 2013 |
| SRR3647355 | BCG | NA | Zhu et al., 2016 |
| ERR2642516 | *M. caprae* | NA | Brites et al., 2018 |
| ERR551023 | *M. caprae* | Congo | Malm et al., 2017 |
| ERR551704 | *M. caprae* | Germany | Malm et al., 2017 |
| ERR552526 | *M. caprae* | Germany | Malm et al., 2017 |
| ERR841382 | *M. caprae* | NA | Domogalla et al., 2013 |
| SRR650219 | *M. caprae* | Germany | Research Center Borstel |
| ERR027294 | *M. microti* | UK | The Wellcome Sanger Institute |
| ERR2659164 | *M. microti* | Italy | Brites et al., 2018 |
| ERR234675 | *M. orygis* | Russia | Casali et al., 2014 |
| ERR2659153 | *M. orygis* | Australia | Brites et al., 2018 |
| SRR3500411 | *M. mungi* | Botswana | Alexander et al., 2016 |
| SRR1239337 | *M. pinnipedii* | Australia | Bos et al., 2014 |
