# Supplementary material for: Independent genomic polymorphisms in the PknH serine threonine kinase locus during evolution of the Mycobacterium tuberculosis Complex affect virulence and host preference
Source: PLoS Pathog. 2020 Dec 21;16(12):e1009061. doi: 10.1371/journal.ppat.1009061 (PMC7785237; doi:10.1371/journal.ppat.1009061)
Supplement: S2 Table — *Gene expression data from M. tuberculosis were obtained from Malone et al. [38]. (DOCX) [file ppat.1009061.s005.docx]

**Table S2. Filtered DE gene overlap between *M.bovis*::pknH^TB^ vs *M. bovis* WT, and *M. bovis* WT vs *M. tuberculosis. **Gene expression data from *M. tuberculosis* were obtained from Malone *et al.* [1].**

| **Tag** | **Gene** | **Category** | **Log_2_FC(*M. bovis* pknH^TB^ vs WT)** | **log_2_FC (*M. bovis* vs *Mtb*)*** |
| --- | --- | --- | --- | --- |
| Mb0173 | *yrbE1A* | virulence, detoxification,adaptation | 1.73 | 1.12 |
| Mb0174 | *yrbE1B* | virulence, detoxification,adaptation | 1.71 | 1.16 |
| Mb0175 | *mce1A* | virulence, detoxification,adaptation | 2.00 | 1.26 |
| Mb0176 | *mce1B* | virulence, detoxification,adaptation | 1.92 | 1.21 |
| Mb0177 | *mce1C* | virulence, detoxification,adaptation | 2.20 | 1.05 |
| Mb0178 | *mce1D* | virulence, detoxification,adaptation | 2.10 | 1.11 |
| Mb0179 | *lprK* | cell wall and cell processes | 2.32 | 1.19 |
| Mb0282 | *Mb0282* | conserved hypothetical protein | 1.48 | -2.16 |
| Mb0718 | *Mb0718* | conserved hypothetical protein | -2.17 | -2.00 |
| Mb0719 | *Mb0719* | conserved hypothetical protein | -2.25 | -1.68 |
| Mb1079 | *Mb1079* | intermediate metabolism and respiration | 1.73 | 1.11 |
| Mb1124 | *desA2* | lipid metabolism | -1.29 | -1.08 |
| Mb1210 | *fdxC* | intermediate metabolism and respiration | -1.59 | -1.05 |
| Mb1576 | *fadD11.1* | lipid metabolism | 2.04 | 1.57 |
| Mb1576 | *fadD11* | lipid metabolism | 2.04 | 3.11 |
| Mb1577 | *plsB1* | lipid metabolism | 2.26 | 1.77 |
| Mb1904 | *Mb1904* | conserved hypothetical protein | -1.79 | 4.04 |
| Mb2076 | *Mb2076* | conserved hypothetical protein | 1.80 | 1.16 |
| Mb2275 | *Mb2275* | intermediate metabolism and respiration | 1.95 | 1.27 |
| Mb2324 | *Mb2324* | conserved hypothetical protein | 1.47 | 1.04 |
| Mb2555 | *vapb17* | virulence, detoxification,adaptation | 1.64 | 1.01 |
| Mb2621 | *fadD9* | lipid metabolism | -1.69 | -1.86 |
| Mb2834 | *Mb2834* | conserved hypothetical protein | -2.13 | -1.47 |
| Mb2836 | *Mb2836* | insertion seqs and phages | -2.73 | -1.73 |
| Mb2898 | *mpb83* | cell wall and cell processes | 2.04 | 5.66 |
| Mb2899 | *dipZ* | intermediate metabolism and respiration | 1.79 | 5.06 |
| Mb2900 | *mpb70* | cell wall and cell processes | 2.32 | 5.93 |
| Mb3389 | *Mb3389* | conserved hypothetical protein | 1.72 | 1.36 |
| Mb3504 | *PE31* | PE/PPE | 2.16 | -2.66 |
| Mb3657 | *Mb3657* | conserved hypothetical protein | 1.98 | 1.43 |
| Mb0173 | *yrbE1A* | virulence, detoxification,adaptation | 1.73 | 1.12 |
| Mb0174 | *yrbE1B* | virulence, detoxification,adaptation | 1.71 | 1.16 |
| Mb0175 | *mce1A* | virulence, detoxification,adaptation | 2.00 | 1.26 |
| Mb0176 | *mce1B* | virulence, detoxification,adaptation | 1.92 | 1.21 |
| Mb0177 | *mce1C* | virulence, detoxification,adaptation | 2.20 | 1.05 |
| Mb0178 | *mce1D* | virulence, detoxification,adaptation | 2.10 | 1.11 |
| Mb0179 | *lprK* | cell wall and cell processes | 2.32 | 1.19 |
| Mb0282 | *Mb0282* | conserved hypothetical protein | 1.48 | -2.16 |
| Mb0718 | *Mb0718* | conserved hypothetical protein | -2.17 | -2.00 |
| Mb0719 | *Mb0719* | conserved hypothetical protein | -2.25 | -1.68 |
| Mb1079 | *Mb1079* | intermediate metabolism and respiration | 1.73 | 1.11 |
| Mb1124 | *desA2* | lipid metabolism | -1.29 | -1.08 |
| Mb1210 | *fdxC* | intermediate metabolism and respiration | -1.59 | -1.05 |
| Mb1576 | *fadD11.1* | lipid metabolism | 2.04 | 1.57 |
| Mb1576 | *fadD11* | lipid metabolism | 2.04 | 3.11 |
| Mb1577 | *plsB1* | lipid metabolism | 2.26 | 1.77 |
| Mb1904 | *Mb1904* | conserved hypothetical protein | -1.79 | 4.04 |
| Mb2076 | *Mb2076* | conserved hypothetical protein | 1.80 | 1.16 |
| Mb2275 | *Mb2275* | intermediate metabolism and respiration | 1.95 | 1.27 |
| Mb2324 | *Mb2324* | conserved hypothetical protein | 1.47 | 1.04 |
| Mb2555 | *vapb17* | virulence, detoxification,adaptation | 1.64 | 1.01 |
| Mb2621 | *fadD9* | lipid metabolism | -1.69 | -1.86 |
| Mb2834 | *Mb2834* | conserved hypothetical protein | -2.13 | -1.47 |
| Mb2836 | *Mb2836* | insertion seqs and phages | -2.73 | -1.73 |
| Mb2898 | *mpb83* | cell wall and cell processes | 2.04 | 5.66 |
| Mb2899 | *dipZ* | intermediate metabolism and respiration | 1.79 | 5.06 |
| Mb2900 | *mpb70* | cell wall and cell processes | 2.32 | 5.93 |
| Mb3389 | *Mb3389* | conserved hypothetical protein | 1.72 | 1.36 |
| Mb3504 | *PE31* | PE/PPE | 2.16 | -2.66 |
| Mb3657 | *Mb3657* | conserved hypothetical protein | 1.98 | 1.43 |
