# Supplementary material for: Independent genomic polymorphisms in the PknH serine threonine kinase locus during evolution of the Mycobacterium tuberculosis Complex affect virulence and host preference
Source: PLoS Pathog. 2020 Dec 21;16(12):e1009061. doi: 10.1371/journal.ppat.1009061 (PMC7785237; doi:10.1371/journal.ppat.1009061)
Supplement: S3 Table — (DOCX) [file ppat.1009061.s006.docx]

**Table S3. Primer sequences used for PCR and qRT-PCR.**

| **Primer** | **Sequence 5’-3’** | **Use** |
| --- | --- | --- |
| pknhdel fw | AAGACCGCTACGCCAGCGC | PCR verification flanking *pknH* deletion |
| pknhdel rv | GCCACTAAGGGCCACGGGTTG | PCR verification flanking *pknH* deletion |
| Pknhdel_2 rv | GTAGGACGGCTTTGGTGCCGG | PCR verification, primer inside *pknH* deletion |
| RT-*pknH*-fw | CTCCTCGGCAGCCACCC | RT-PCR verification of *pknH^TB^* expression |
| RT-*pknH*-rv | CTTTGGTGCCGGCTGGACAC | RT-PCR verification of *pknH^TB^* expression |
