# Supplementary material for: Independent genomic polymorphisms in the PknH serine threonine kinase locus during evolution of the Mycobacterium tuberculosis Complex affect virulence and host preference
Source: PLoS Pathog. 2020 Dec 21;16(12):e1009061. doi: 10.1371/journal.ppat.1009061 (PMC7785237; doi:10.1371/journal.ppat.1009061)
Supplement: Attachment — Submitted filename: POINT-BY-POINT REPLY LETTER.pdf [file ppat.1009061.s007.pdf]

Point-by-point response to reviewers' comments to PPATHOGENS-D-20-01575

We appreciate the helpful comments and constructive criticisms of the reviewers, which we feel have contributed to substantial revision of our manuscript. We trust we were able to respond to their suggestions and concerns, meeting their expectations.

Major Issues: Key Experiments Required for Acceptance

Reviewer #1: No response

Reviewer #2:

1) Reviewer's comment: The mouse infection experiments are also convincing as there is a > than 1 log CFU difference shown betweenis strains and M. tuberculosis strains, as well as between M. bovis parental strains and pknH knock in mutants. However, one point should be better explained by the authors, how they ascertained that the initial dose that was used to infect mice is comparable between strains, as there are no day 1 data shown.

Author's response: In our laboratory, we have been using intranasal administration for several years, and we have published different studies using this route to deliver pulmonary Mtb strains. Indeed, in the past we used to evaluate lung CFUs at day 1 following intranasal administration (this is shown for instance in the supplementary material of Aguilo *et al*, NatComm, 2017, https://doi.org/10.1038/ncomms16085). However, since this procedure is well optimized in our lab, with highly reproducible results, we stopped doing this control with the aim to reduce the number of mice.

Thus, to ascertain the initial dose of bacteria in the lungs following intranasal delivery, we normally determine the CFUs from the bacterial suspension that we use for mouse infection. We have clarified this point in the revised methods section (lines 608-609).

2) Reviewer's comment: For the discussion, it would be helpfull if the authors could better explain whether the M. bovis strains used in the mouse experiments expressed the two copies of pknH they contain, or if the two genes were not expressed ?. This was not clear from the reading and it would help to better understand the rational of adding the pknH gene from M. tuberculosis.

Author's response: *pknH* genes are expressed in the *M. bovis* wild type strains under exponential culture conditions. This is illustrated in the figure 1 of this letter, in which we analyzed *pknH* expression from cDNA of *M. bovis* AF2122 and AN5 WT and knock-in strains, using a primer pair flanking the proline-rich region (in the figure S1 from original manuscript we showed a PCR where one of the primers was inside the proline-rich region and therefore the PCR was negative for the *M. bovis* wild-type strains). The result described in figure 1 shows that *pknH* is expressed in both *M. bovis* WT strains. In the *M. bovis* knock-in strains two amplifications products were obtained, corresponding to the expression of the *M. bovis pknH* genes and of the introduced *M. tuberculosis pknH* gene. This result has been included as a new panel of the figure S1.

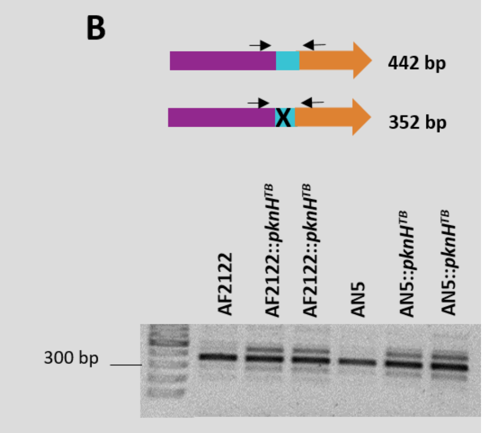

Figure 1. PCR from M. bovis cDNA using *pknH* proline-rich region flanking primers

This PCR can not discriminate between *pknH1* and *pknH2* expression in the *M. bovis* strains. However previously published RNA-seq data of the *M. bovis* reference strain AF2122 revealed that both *pknH1* and *pknH2* genes are expressed under exponential *in vitro* culture (1). In this work we saw that *pknH1*, *pknH2* and *tbd2* were expressed by *M. bovis* under exponential *in vitro* culture in Sauton's media by using RNA-seq (191.55±23.12 TPM, 66.33±3.18 TPM, and 133.26 ±12.25 TPM respectively, n=6 replicates) (1).

Although the two *pknH* copies seem to be expressed in *M. bovis,* both of them have a deletion in the proline-rich region, so there is not a full copy of this gene expressed by any of the *M. bovis* strains analyzed, in contrast to the other MTBC such as *M. tuberculosis*. Thus, the rational of adding the *M. tuberculosis pknH* gene was to introduce a complete *pknH* gene in *M. bovis*, since we hypothesized that this deletion may affect PknH function, and that the absence of a complete *pknH* gene could impact bacterial virulence. This point has been clarified in the revised manuscript (lines 445-450).

3) The RNA seq data should also be explained in more detail. It is not clear why an increased expression of espACD in the pknH TB knock in strains should cause decreased virulence, especially as the regulation of the espACD operon is complex in M. tuberculosis (PhoPR, EspR, etc) and different in M. bovis (RD8 etc). The role of pknH in this regulation process should be hypothesized.

Author's response: In the revised version we have extended the description of the RNAseq data. Indeed, following reviewer advise we have included a specific section of the RNAseq data (lines 284-333).

We agree with the reviewer in the apparent contradiction of this result, since *espACD* has been shown to be essential in ESX-1 secretion, a key determinant of *M.* tuberculosis virulence. In the context of the PhoP/PhoR two-component systems, *phoPR* mutants has shown a reduction of *espACD* expression which leads to reduced virulence. However, it has also been previously shown that upregulation of *espACD* does not always lead to increased ESX-1 secretion or increased virulence. Pang et al (2) showed that while deletion of MprAB in *M. tuberculosis* led to increased transcription of *espA* and *espD*, there was a concomitant decrease in EspA, EspB and EsxA secretion. It is well accepted that EspACD proteins must be secreted through ESX-1 to trigger their virulence mechanism. Thus, a more detailed description of the linkages between PknH and the ESX-1 system in *M. bovis* should be done to clarify our observations.

This explanation has been added to the revised discussion section (lines 475-489), together with the reference of Pang et al.

Minor Issues: Editorial and Data Presentation Modifications

Reviewer #1:

1) Particularly where the author's make the claim that there is variability within the same lineage regarding the structure of the pknH locus, it would have been preferable to have confirmed some of the gene arrangements via PCR and/or Sanger sequencing (or cloning and sequencing) rather than relying solely on alignments generated from someone else's WGS data. As the author's themselves have highlighted, recombination and repetitive sequences in this variable region have previously led to annotation errors (and confusion) for various MTBC members.

Author's response: In order to avoid possible annotations errors from previous studies, we did not use annotations based on existing assemblies since misannotations are likely present in this locus, due to the fact that homologous domains shared between *pknH* genes cannot be uniquely aligned with short reads. Such reads are marked as multi-mappers and ignored; resulting assemblies are therefore split into contigs over such a region and annotation software may miss genes. To avoid this, we downloaded the original raw sequence reads in fastq format from GenBank for each available isolate (Table S1). The raw reads were aligned to a reference sequence (*M. africanum* GM041182 RD900 locus) representing the most complete version of the RD900 across the MTBC. In this way we could most readily see missing elements in the other species. Reads were aligned using bwa (mem algorithm) (3) and the resulting bam files were sorted and indexed using samtools. The alignments were visualized in IGV (4) to identify the presence or absence of RD900 and the presence of the deletion in the proline-rich region within *pknH* genes. Finally, a variant calling analysis was performed to identify point mutations present in this region from the different MTBC analysed species. Variant calling, filtering and consequence calling were performed using freebayes (https://arxiv.org/abs/1207.3907) and bcftools.

In agreement with the reviewer, we think this point was not properly explained in the original manuscript, so we have included this explanation in the revised methods section (lines 655-673).

2) In regards to the RNAseq analysis comparing M. bovis with M. bovis::mtb-pknH (Fig. 4a) – are the author's aware if any of the genes that appear in their list of the "top DE genes" are also variably expressed between, for example, M. canettii or M. tuberculosis when compared to M. bovis?

Author's response: An analysis of differentially-expressed (DE) genes between *M. bovis* AF2122/97 and *M. tuberculosis* H37Rv during in vitro growth has been previously described (5). Thus, to address this question of the reviewer we have examined whether introduction of $pknH^{TB}$ into *M. bovis* switched the expression of these DE genes in *M. bovis* to more closely match their expression in *M. tuberculosis* H37Rv. This revealed a minimal overlap, with only *Mb1904, Mb3504* and *PE31* showing expression in AF2122::$pknH^{TB}$ that more closely resembled levels in *M. tuberculosis* H37Rv than *M. bovis* WT. These results are comented in the revised manuscript (lines 323-333 and 490-495)). A new table compiling these data has been included in the supplementary material (Table S2).

1) The manuscript should be proofread by a native English speaker, as there are several grammar mistakes apparent, e.g. line 463.

Author's response: The revised manuscript has been revised by a native English speaker.

2) The nomenclature of pknHTB gene is confusing, "TB" could be noted as superscript or in a similar fashion.

Author's response: Done

3) A seperate paragraph with heading should be written for the RNA seq results.

Author's response: Done (line 284)

4) It would be interesting to read the authors opinion on the role of the tbd2 gene present in RD900.

Author's response: We agree with the reviewer that it would be interesting to include the available data about the *tbd2* gene. The *tbd2* gene encodes a transmembrane ATP binding cassette (ABC) transport protein of unknown function that has a central ATP binding domain and six possible membrane-spanning domains in the C-terminal portion (6). The N-terminal region contains a FHA domain, which is a phosphoprotein recognition domain. Thus, it can be hypothesized that Tbd2 could be phosphorylated by the adjacent PknH protein or by others STPKs, as phosphorylation of other PknH substrates, such as EmbR, has been shown to be mediated by the FHA domain (7). This point has been clarified in the revised manuscript.

We have included this explanation in the revised section (lines 408-416)

5) In reaction to a previous paper on the virulence of M. bovis and M. tuberculosis, interesting comments were written on the ability or inability of certain MTBC members to spread in certain hosts (PMID: 25174642; PMID: 25435136) which might also apply to this work, and which should be discussed

Author's response: We agree with the reviewer that we have not discussed enough the

impact of our observations in the context of the ability of MTBC members to spread into different hosts. We have added the following two paragraphs to the revised discussion section (lines 530-554).

Previous studies have demonstrated higher virulence of *M. bovis* compared to *M. tuberculosis* in several animal models, such as mice or rabbits. In addition, experimental infections of cattle have demonstrated that *M. tuberculosis,* at least from lineage 4, is attenuated in the bovine host (8, 9). Clearly, as we show here, the genetic differences between *M. bovis* and *M. tuberculosis* can contribute to the varying degrees of virulence shown for these animal hosts. Ultimately however, an evolutionary successful strategy depends on host factors as well as pathogen virulence, the interaction of which drives transmission and maintenance of the pathogen in the host population. Indeed, *M. bovis* is able to spread and sustain across different animal populations, both domesticated (e.g. cattle) and wild (e.g. badgers and deer), yet human-to-human transmission of *M. bovis* is a rare event. Thus one could hypothesize that *M. bovis* has evolved to exploit dissemination routes distinct from respiratory transmission. Conversely, *M. tuberculosis* has evolved towards human-adaptation to which respiratory dissemination and a functional PknH are integral.

In this regard, the two component PhoPR system seems to have a crucial role. *M. bovis* strains contain point mutations in the region encoding the extracellular domain of PhoR, which impairs the signalling of this system. Thus, as described by Gonzalo-Asensio et al, the expression of *phoP* regulon genes is divergent in *M. bovis* as compared to *M. tuberculosis*, which could again explain their compromised capacity to spread via human-to-human. Interestingly, an *M. bovis* strain, called the 'B strain' caused a severe TB outbreak in Spain in the 1990s and disseminated efficiently among humans. This latter strain overexpressed the *phoP* gene and subsequently expressed the PhoP regulon at levels similar to *M. tuberculosis*, highting the linkages between this regulon and efficient huma-to-human transmission.

REFERENCES

1. Doctoral Thesis. Malone KM. An integrative 'omics approach to define functional variation between the human and bovine tubercle bacilli. p.69: University College Dublin. School of Veterinary Medicine. ; 2016. https://researchrepository.ucd.ie/bitstream/10197/7539/3/Kerri_Malone_thesis_final_Mar16.pdf

2. Pang X, Samten B, Cao G, Wang X, Tvinnereim AR, Chen XL, et al. MprAB regulates the espA operon in Mycobacterium tuberculosis and modulates ESX-1 function and host cytokine response. Journal of bacteriology. 2013;195(1):66-75.

3. Li H, Durbin R. Fast and accurate short read alignment with Burrows-Wheeler transform. Bioinformatics. 2009;25(14):1754-60.

4. Robinson JT, Thorvaldsdottir H, Winckler W, Guttman M, Lander ES, Getz G, et al. Integrative genomics viewer. Nature biotechnology. 2011;29(1):24-6.

5. Malone KM, Rue-Albrecht K, Magee DA, Conlon K, Schubert OT, Nalpas NC, et al. Comparative 'omics analyses differentiate Mycobacterium tuberculosis and Mycobacterium bovis and reveal distinct macrophage responses to infection with the human and bovine tubercle bacilli. Microbial genomics. 2018;4(3).

6. Bentley SD, Comas I, Bryant JM, Walker D, Smith NH, Harris SR, et al. The genome of Mycobacterium africanum West African 2 reveals a lineage-specific locus and genome erosion common to the M. tuberculosis complex. PLoS neglected tropical diseases. 2012;6(2):e1552.

7. Molle V, Kremer L, Girard-Blanc C, Besra GS, Cozzone AJ, Prost JF. An FHA phosphoprotein recognition domain mediates protein EmbR phosphorylation by PknH, a Ser/Thr protein kinase from Mycobacterium tuberculosis. Biochemistry. 2003;42(51):15300-9.

8. Villarreal-Ramos B, Berg S, Whelan A, Holbert S, Carreras F, Salguero FJ, et al. Experimental infection of cattle with Mycobacterium tuberculosis isolates shows the attenuation of the human tubercle bacillus for cattle. Sci Rep. 2018;8(1):894.

9. Whelan AO, Coad M, Cockle PJ, Hewinson G, Vordermeier M, Gordon SV. Revisiting host preference in the Mycobacterium tuberculosis complex: experimental infection shows M. tuberculosis H37Rv to be avirulent in cattle. PloS one. 2010;5(1):e8527.
